# Harnessing Oleanolic Acid and Its Derivatives as Modulators of Metabolic Nuclear Receptors

**DOI:** 10.3390/biom13101465

**Published:** 2023-09-28

**Authors:** Mohamed O. Radwan, Sultan F. Kadasah, Salha M. Aljubiri, Abdulmajeed F. Alrefaei, Mahmoud H. El-Maghrabey, Mohamed A. El Hamd, Hiroshi Tateishi, Masami Otsuka, Mikako Fujita

**Affiliations:** 1 Medicinal and Biological Chemistry Science Farm Joint Research Laboratory, Faculty of Life Sciences, Kumamoto University, Kumamoto 862-0973, Japan; htateishi@kumamoto-u.ac.jp (H.T.); motsuka@gpo.kumamoto-u.ac.jp (M.O.); mfujita@kumamoto-u.ac.jp (M.F.); 2 Department of Biology, Faculty of Science, University of Bisha, Bisha 61922, Saudi Arabia; sukadasah@ub.edu.sa; 3 Department of Chemistry, College of Science, University of Bisha, Bisha 61922, Saudi Arabia; sragh@ub.edu.sa; 4 Department of Biology, Umm Al-Qura University, Makkah 21955, Saudi Arabia; afrefaei@uqu.edu.sa; 5 Department of Pharmaceutical Analytical Chemistry, Faculty of Pharmacy, Mansoura University, Mansoura 35516, Egypt; dr_m_hamed@mans.edu.eg; 6 Department of Pharmaceutical Sciences, College of Pharmacy, Shaqra University, Shaqra 11961, Saudi Arabia; 7 Department of Pharmaceutical Analytical Chemistry, Faculty of Pharmacy, South Valley University, Qena 83523, Egypt; 8 Department of Drug Discovery, Science Farm Ltd., Kumamoto 862-0976, Japan

**Keywords:** oleanolic acid, nuclear receptors, metabolic disorders, NASH, farnesoid X receptor, liver X receptor, peroxisome-proliferator activated receptors

## Abstract

Nuclear receptors (NRs) constitute a superfamily of ligand-activated transcription factors with a paramount role in ubiquitous physiological functions such as metabolism, growth, and reproduction. Owing to their physiological role and druggability, NRs are deemed attractive and valid targets for medicinal chemists. Pentacyclic triterpenes (PTs) represent one of the most important phytochemical classes present in higher plants, where oleanolic acid (OA) is the most studied PTs representative owing to its multitude of biological activities against cancer, inflammation, diabetes, and liver injury. PTs possess a lipophilic skeleton that imitates the NRs endogenous ligands. Herein, we report a literature overview on the modulation of metabolic NRs by OA and its semi-synthetic derivatives, highlighting their health benefits and potential therapeutic applications. Indeed, OA exhibited varying pharmacological effects on FXR, PPAR, LXR, RXR, PXR, and ROR in a tissue-specific manner. Owing to these NRs modulation, OA showed prominent hepatoprotective properties comparable to ursodeoxycholic acid (UDCA) in a bile duct ligation mice model and antiatherosclerosis effect as simvastatin in a model of New Zealand white (NZW) rabbits. It also demonstrated a great promise in alleviating non-alcoholic steatohepatitis (NASH) and liver fibrosis, attenuated alpha-naphthol isothiocyanate (ANIT)-induced cholestatic liver injury, and controlled blood glucose levels, making it a key player in the therapy of metabolic diseases. We also compiled OA semi-synthetic derivatives and explored their synthetic pathways and pharmacological effects on NRs, showcasing their structure-activity relationship (SAR). To the best of our knowledge, this is the first review article to highlight OA activity in terms of NRs modulation.

## 1. Introduction

Nuclear receptors (NRs) are ligand-activated transcription factors encoded by 48 genes in humans and classified into seven subfamilies [1]. They are located inside the cell and comprise the receptors for steroid hormones, lipophilic vitamins, sterols, and cholesterol metabolites [2]. NRs play a pivotal role in biological processes, including development, inflammation, metabolism, and reproductive health [1,2,3,4,5,6]. NRs dysregulation is linked to a vast array of diseases; hence, they represent attractive druggable targets considering their possible modulation with small molecules, accounting for billions of dollars in annual pharmaceutical sales [1,2,4,7,8]. Approximately half of NR are classified as orphan receptors since they do not have well-characterized endogenous or synthetic ligands [2,9,10,11,12]. Most ligands for nuclear receptors are small, lipophilic molecules that can easily penetrate the cell membrane and modulate their corresponding receptors [4]. Some NRs, such as glucocorticoid receptors (GR; NR3C1), translocate from the cytoplasm to the nucleus in case of ligand binding; others reside in the nucleus even in the absence of cognate ligands.

Most NRs function as dimers, either homodimers, such as steroid receptors including GR, androgen receptors (AR; NR3C4), mineralocorticoid receptors (MR; NR3C2), and estrogen receptors (Erα; NR3A1 and Erβ; NR3A2) [13,14], or heterodimers with retinoic acid X receptor α (RXRα; NR2B1) as a common obligatory partner [15]. Examples of NRs that form heterodimers are farnesoid X receptors (FXRα; NR1H4), peroxisome-proliferator activated receptors (PPARα; NR1C1, PPARβ; NR1C2, and PPARγ; NR1C3), liver X receptors (LXRα; NR1H3 and LXRβ; NR1H2), retinoic acid receptors (RARα; NR1B1, RARβ; NR1B2, and RARγ; NR1B3), pregnane X receptors (PXR, NR1I2), thyroid hormone receptors (THRα, NR1A1, and THRβ, NR1A2), and vitamin D receptors (VDR, NR1I1) [1]. The heterodimer is either permissive, which can be activated by RXR’s or its partner’s ligand, or nonpermissive, which is activated only by the partner’s ligand. This confers RXRα a special importance among NRs [16]. On the other hand, retinoic-acid-receptor-related orphan receptors (RORα; NR1F1, RORβ; NR1F2, and RORγ, NR1F3) work as monomers or homodimers [17].

**Figure 1:**
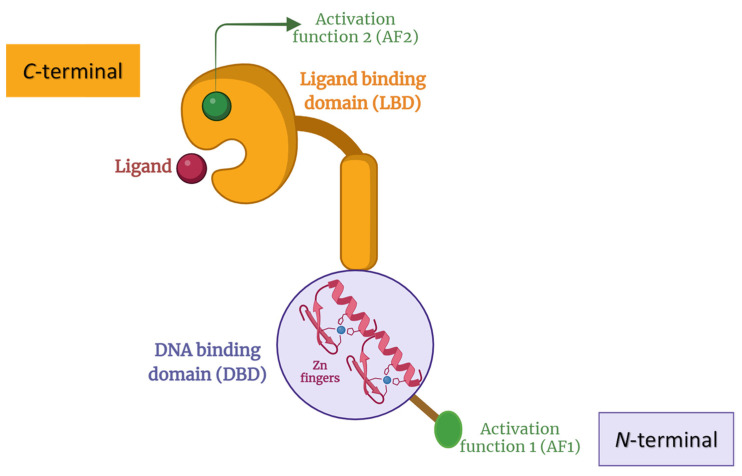
Schematic representation of NRs structure with AF-1 region followed by a highly conserved DBD with two zinc fingers. This is linked to a less conserved and short hinge region followed by LBD and AF-2.

The typical structure of NRs is shown in Figure 1. The N-terminal has a ligand-independent activation function (AF1) connected to a highly conserved DNA binding domain (DBD) with two zinc fingers and is rich in cysteines and basic amino acids. The flexible hinge region is short and with a low degree of conservation [2]. The ligand binding domain (LBD) contains a pocket for endogenous ligands and a ligand-dependent activation factor (AF-2) ending by a highly variable C-terminal [2,18]. The ligand-binding pocket is the least conserved region on LBD, which makes it the main target for NRs modulation in terms of medicinal chemistry [19,20,21]. In the presence or absence of ligands, most NR can recruit a specific cofactor, recognize specific DNA-response elements in the promoters of their cognate target genes, and modify their ability to recruit a range of other transcriptional proteins that affect gene expression rate [2]. For example, THR stops target gene transcription in the absence of thyroid hormone and vice versa.

It is noteworthy that the resulting NR modulation occurs in a tissue-specific manner depending on the type of recruited cofactor, which makes it more complex to obtain a selective NR modulator. For example, tamoxifen works as an ER antagonist in breast tissue but as an agonist in the uterus and bone, which may trigger uterus cancer. A better selective modulator is raloxifene, which is an agonist of bone ER but an antagonist for both breast and uterus ER, making it a safer choice with better clinical outcomes than tamoxifen for hormonal breast cancer therapy [1,2,4].

Natural products are privileged structures and rich sources of approved pharmaceutical products [22,23]. Pentacyclic triterpenes (PTs) are bio-nutrient phytochemicals present in higher plants and endowed with ubiquitous bioactivities [24,25,26,27,28,29,30]. Their lipophilic nature confers them affinity to fit into NRs LBD, which is basically activated by endogenous lipophilic ligands. They are mainly divided into four chemical scaffolds, namely, oleanane, ursane, lupane, and friedelane [24]. Oleanolic acid (OA), 3-beta-Hydroxyolean-12-en-28-oic acid, is a common oleanane type PT that has been widely studied in terms of medicinal chemistry and bioactivity owing to its multitude of health benefits. The most important sources of OA in the human diet are olives (O. europaea L.), from which the compound derives its name [31], followed by various legumes, jujube, ginseng, wild sage, and hawthorn berries. The literature is loaded with plenty of success stories linking oleanolic acid and its derivatives with treatments for diverse ailments. OA and its derivatives were reported to suppress the proliferation of hepatocellular carcinoma [32,33], lung cancer [34], colon cancer [35], human bladder cancer [36], breast cancer [37], and leukemia [38]. OA alters different cellular pathways implicated in cancer [31,39]. Alongside its anticancer potential, OA has a broad hepatoprotective [25,40,41,42,43,44], antiatherosclerosis [45,46], and antidiabetic activity [47,48].

**Figure 2:**
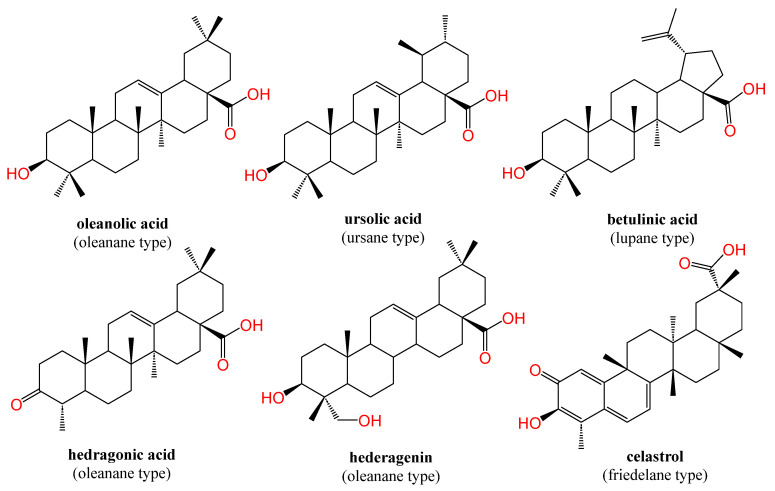
Chemical structure of some PTs from oleanane, ursane, lupane, and friedelane types as NRs modulators. Atoms were colored by element.

De facto, many natural products of both plant and marine origin have been proven effective with respect to NRs modulation [18,19,20,49,50,51,52]. Theonellasterol is a natural sterol from a marine sponge with an FXR antagonistic effect and protective properties against cholestasis-induced liver injury [53]. Guggulsterone is a natural phytosteroid and a promiscuous NRs modulator that is widely used as a positive control in NRs-related assays. Of special interest, PTs proved to be emerging NRs ligands with a plethora of therapeutic benefits. Ursolic acid was identified as an RORγt inverse agonist [54] in addition to LXRα antagonist [55]. Betulinic acid was able to attenuate non-alcoholic steatohepatitis (NASH) and liver endoplasmic reticulum stress in mice models via FXR activation [56]. Hedragonic acid, an oleanane type PT, isolated from *Celastrus orbicalatus* Thunb., was identified as an effective and selective FXR agonist over other metabolic NRs with hepatoprotective properties against paracetamol-induced injury. Indeed, hedragonic acid was co-crystalized with FXR (protein data bank accession code: 5WZX) [57]. Its congener, hederagenin, upregulated the expression of FXR in colonic epithelial cells T84 and promoted agonist-induced FXR signaling, which can play a role in the treatment of intestinal tumorigenesis and diarrhea [58,59]. Celastrol-induced modulation of Nur77 nuclear receptor holding a clinical promise for inflammation therapy [60]. OA and other examples of PTs with NRs modulation effect are depicted in Figure 2, indicating the chemical type of each one.

In a similar vein, among PTs, OA garnered the highest interest as a ligand of nuclear receptors with potential application in the treatment of metabolic diseases. This provoked us to compile and summarize the previous results in a review article that would be a cornerstone for future studies on NRs modulation by PTs and OA as a representative. There are different reviews on OA highlighting its ubiquitous bioactivities [31,39,61]; having said that, none of them discusses its pharmacodynamic effects on NRs.

## 2. Methodology

We used the keywords pentacyclic triterpenes or oleanolic acid and nuclear receptors for our search in the Web of Science database. This resulted in approximately 110 research articles, review articles, and patents, of which 70 were considered for this review. The other articles were excluded as they mainly focused on other types of receptors or other PTs.

## 3. Oleanolic Acid and Its Derivatives as Nuclear Receptors’ Modulators

### 3.1. Modulation of FXR

FXR is implicated in bile acid, lipid, and glucose homeostasis, and hepatic inflammation, regeneration, and fibrosis, and is widely distributed in organs such as the liver, kidney, intestinal tract, and adrenal gland [62,63,64,65]. Bile acids, the natural ligands of FXR, have been considered potential intestinal tumor promoters [66]. FXRα is an attractive drug target for the treatment of diverse metabolic diseases, including NASH, primary biliary cirrhosis (PBC), diabetes [67], and atherosclerosis [5,67,68,69,70] in addition to cancer [71]. Interaction with FXRα recruits coactivators such as SRC1 or corepressors such as nuclear receptor corepressor (NCoR1) [5]. Extensive research effort ended up with the discovery of some clinical FXR ligands, including obeticholic acid, the only approved FXR modulator to date for PBC therapy, and under clinical phase III for the treatment of NASH [72].

FXR expression is found to be decreased in human intestinal tumors due to the promotion of Wnt signaling, while the reactivation of FXR in a xenograft model via adenoviral infection induced cytotoxicity through the induction of apoptosis and inhibition of proinflammatory and antiapoptotic genes [59]. On the contrary, FXR activation promotes transforming growth factor β (TGF-β) induced epithelial-mesenchymal transition in hepatocellular carcinoma cells [73]. By virtue of its complicated role in cholesterol and bile acids homeostasis, both FXR agonists and antagonists may be therapeutically useful for the treatment of metabolic diseases [5].

The first report relating OA with FXR was disclosed by Liu and Wong in 2010 [74]. They supposed that OA health benefits are partially attributed to FXR modulation. Luciferase assay using hepatocellular carcinoma cells showed that OA competitively suppressed the activity of FXR-LBD induced by its endogenous activator chenodoxycholic acid (CDCA) without affecting the latter’s metabolism. OA not only bound to FXR-LBD and suppressed its activity in a dose-dependent manner, but also partially blocked the interaction with the coactivator SRC-3, as shown in a cell-free model. At 25 μM concentration of OA in HepG2, quantitative RT-PCR (RT-qPCR) showed that OA partially blocked CDCA induction of bile salt export pump (BSEP) and cytochrome P450 7A1 (CYP7A1) target genes but did not affect the expression level of another target gene, organic solute transporter (OST-β), and slightly enhanced SHP, suggesting that OA works as a gene selective modulator of FXR. OA did not significantly reduce LXRα and LXRβ activity induced by their known synthetic ligand TO901317 [74,75].

Another interesting work showcased the effect of OA on mice models with obstructive cholestasis by bile duct ligation (BDL) [76]. Basically, the histological examination of hepatocytes indicated that 20 mg/kg OA administration ameliorates BDL-induced liver injury. Furthermore, pretreatment with OA or ursodeoxycholic acid (UDCA), the only approved drug for treatment of PBC by Food and Drug Administration (FDA), in BDL mice lowered the level of alanine aminotransferase (ALT) by 59% and 41%, aspartate transaminase (AST) by 33% and 28%, alkaline phosphatase (ALP) by 44% and 39%, respectively. Furthermore, OA attenuated BDL-induced extrahepatic cholestasis in association with association with enhancement of urine bile acid output. Mechanistically, gene expression analysis proved that OA resulted in increased mRNA expression of bile acid efflux transporters MRP2, MRP3, and MRP4 [44], which is ascribed to OA-mediated accumulation of nuclear factor-erythroid 2-related factor (NRF2). A significant decrease in Bsep expression when OA was administrated to Sham mice was also further confirmed by RT-qPCR. The latter effect was confirmed to be due to OA antagonism of FXR through a dual luciferase reporter assay in HepG2 cells. In the latter assay, CDCA was used as a positive control and significantly enhanced the luciferase activity of the FXR reporter gene, which was opposed by co-treatment with OA in a dose-dependent manner. Taken together, Chen et al. concluded that OA’s protective role against BDL-induced extrahepatic cholestasis is ascribed to increasing basolateral bile acid export, probably via NRF2-mediated upregulation of MRP2, MRP3, and MRP4; meanwhile, decreased canalicular Bsep expression by OA, which is mediated by FXR antagonism, may also have a paramount role in attenuating bile duct injury [43,44,76].

**Figure 3:**
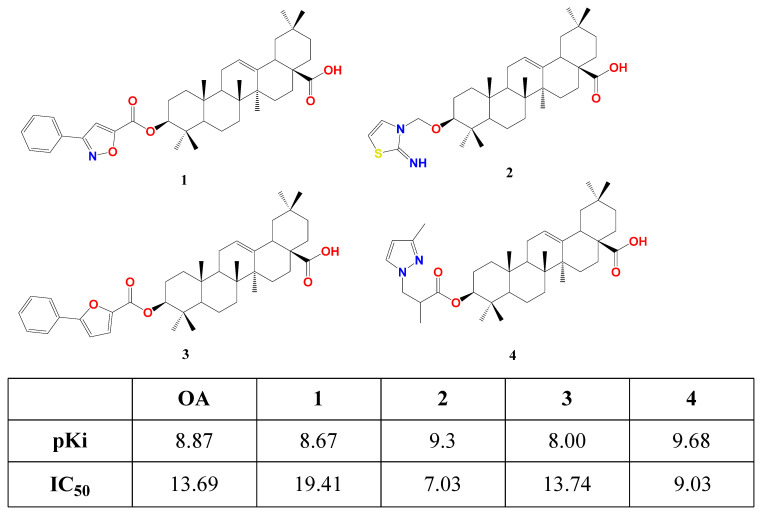
Structure of 3β esters of OA derivatives **1**–**4** showing their theoretical (pKi) and experimental effect (IC_50_) on FXR. Atoms were colored by element [77].

Fang’s research group succeeded in affording the first semi-synthetic OA derivatives as FXR antagonists in 2017 [77]. They designed and synthesized four OA derivatives through the transformation of its 3β-OH, considering the docking score of the candidates versus fexaramine as a control. They used the crystal structure of FXR as a model (PDB code: 1OSH), utilized Autodock 4.2 software in computational work, and synthesized the four top-scored compounds **1**–**4**. These esters were afforded through reacting free OA or protected benzyl OA ester with the appropriate activated acid, followed by catalytic hydrogenolysis for deprotection. In human embryonic kidney 293T cells, compounds **1**–**4** suppressed FXR transactivation in a concentration-dependent manner with respective IC_50_ 19.41, 7.03, 13.74, and 9.03 μM as settled by a dual-luciferase reporter assay. This is in agreement with their predicted docking energy values pKi (Figure 3). Seemingly, **2** and **4** have similar placement in FXR-LBD, forming two T-shaped π-π stacking with Trp458 in helix 11. This crucial interaction is missing in the case of OA, **1**, and **3** [77].

Intriguingly, Pan et al. revealed that OA is likely to work as an FXR agonist. They studied the OA effect in human umbilical vein endothelial cells HUVECs atherosclerosis model by treating them with oxidized low-density lipoprotein (ox-LDL, 100 μg/mL) for 24 h [78]. Basically, OA interrupted the ox-LDL-induced cell apoptosis in the HUVECs. Luciferase reporter assay revealed that the FXR relative luciferase activity was significantly higher in the case of OA treatment in a dose-dependent manner, leading to angiotensin (Ang)-(1–7) upregulation, which, in turn, perturbs the development of atherosclerosis. The results were further validated in New Zealand white (NZW) rabbits as an atherosclerosis animal model. The atherosclerosis assessment at the abdominal aorta and thoracic aorta of the animal models was performed by histopathological analysis in the presence of OA and simvastatin as a positive control. Both OA and simvastatin inhibited the development of atherosclerosis by minimizing the aortic lesion area size and enhancing the collagen content. Although this study is in discrepancy with previous studies that reported FXR antagonism by OA, it introduced an insight into the therapeutic potential of the latter for the treatment of atherosclerosis [78]. It is worth repeating that the NRs modulation is tissue-specific; therefore, it is quite normal to find different pharmacodynamics in different tissues.

Another study explored the underlying mechanism of OA in alleviating alpha-naphthol isothiocyanate (ANIT)-induced cholestatic liver damage in rats instead of BDL [79,80]. As anticipated, OA decreased hepatocyte necrosis and reduced inflammatory cell infiltration in a similar way to UDCA. In rat hepatocytes, OA significantly restored glutathione levels of rat primary hepatocytes reduced by ANIT. This is by reversing the high serum levels of ALP, ALT, AST, total bile acid and (TBA), total bilirubin (TBiL), and gamma-glutamyl transferase (γ-GT) levels in the ANIT-induced model as shown by RT-qPCR. This is attributed to the restoration of FXR and Nrf2 mRNA and protein levels, which were reduced in ANIT. Consequently, treatment with OA decreased the expression of Cyp7a1 mRNA and protein in rats and restored Bsep levels [81].

The same research group conducted an extensive mechanistic study [82]. They compared OA protective effect on ANIT-induced cholestatic liver injury in wild-type and Nrf2 gene knockdown rats and demonstrated that the effect was much weaker in the latter case. This highlights the important role of OA in stimulating the Nrf2 pathway. Likewise, the protective effect of OA against ANIT-induced cholestatic liver injury was relatively weaker in FXR knockdown than in type rats. This demonstrates that the protective effects of OA on ANIT-induced injury and its regulatory role of the bile acids homeostasis gene are mainly ascribed to the simultaneous activation of NRF2 and FXR dual signaling pathways. The authors found a correlation between NRF2 and FXR signaling [82].

By virtue of the cellular context effect of NRs modulators, Fallon et al. studied the effect of hederagenin and OA on FXR in human colonic epithelial cells T84 in comparison to the GW4064, a synthetic FXR agonist. Surprisingly, they found that both hederagenin and OA compounds do not have direct agonistic FXR activity in this model. Having said that, they induced the overexpression of FXR mRNA and protein and upregulated GW4064-induced FXR signaling. This opens the way for the potential application of OA in colon cancer and secretory diarrheas [58].

The same research group of Fang reported a class of 12β-oxygenated oleanolic alkyl esters with FXR modulation properties [83]. In brief, OA 28-COOH was protected by benzylation, and 3-OH was protected by etherification with *t*-butylmethylsilyl (TBS). Olefin of the double-protected derivative was oxidized by *meta*-chloroperoxybenzoic acid (*m*-CPBA), affording a 12-oxo derivative. The latter was reduced by sodium borohydride (NaBH_4_) to a mixture of 12β- and 12α-hydroxyl derivatives in ca. 2:1 ratio. The 12β-OH compound was reacted with the corresponding carboxylic acids in the presence of N,N’-diisopropylcarbodiimide (DIC) or 1-(3-dimethylaminopropyl)-3-ethylcarbodiimide hydrochloride (EDCI) and 4-dimethylaminopyridine (DMAP), followed by deprotection of TBS and Bn groups with BF_3_.Et_2_O and Pd/C hydrogenolysis, respectively, to furnish a series of 12-OA alkyl esters. Various aliphatic and aromatic substituents were used with different polarities.

**Figure 4:**
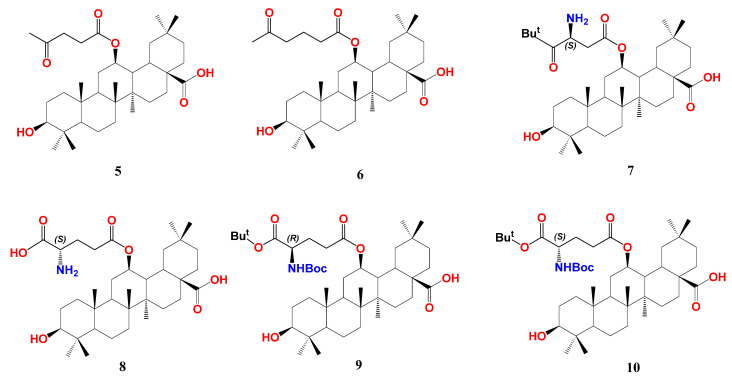
Structure of 12β-esters of OA (5—10) [83,84] p. Atoms were colored by element.

In HEK293T cells, screening of the synthesized series at 10 μM showed that compound **5** with an acetopropionyl group and **6** with a 4-acetobutanoyl group of the 12-*O*-alkanoic acid esters are pronounced FXR antagonists opposing the CDCA effect without observable cytotoxicity at 10 µM (Figure 4). Compounds with carboxy-, amino-, or phenyl terminal groups demonstrated less activity. Owing to its prominent antagonistic activity at 10 and 1 µM, the IC_50_ of compound **6** was calculated to be 0.10 µM. Its binding mode and pharmacophore placement into FXR-LBD is comparable to the FXR antagonist, ivermectin, as calculated by docking simulation [51]. To assess its selectivity, the authors tested compound **6** for the action on an array of NRs, including RXRα, RXRβ, RXRγ, PPARα, PPARβ, PPARγ, LXRα, LXRβ, PXR, and another bile acid receptor GPBAR compared to guggulsterone. While the latter antagonizes almost all tested NRs, compound **6** demonstrated an outstanding antagonism (>90%) against FXR and inconsiderable antagonism (10–20%) against LXRα and PPARα.

Transcription of FXR downstream genes was then evaluated in HepG2 cells. An amount of 10 μM of **6** efficiently reversed the induction of small heterodimeric partner (SHP) and BSEP by 50 μM of the endogenous agonist CDCA without significant effect on sterol regulatory element-binding protein-1c (SREBP-1c and CYP7A1). This is controversial due to the discrepancy with the effect of the parent OA, which suppresses CYP7A1 expression [74]. Intriguingly, in the absence of CDCA, compound **6** clearly hindered SHP, BSEP, and CYP7A1 at 10 μM but did not affect the expression of SREBP-1c, revealing a unique FXR downstream regulation.

Upon exploring the possible effect of **6** on FXR-controlled genes that regulate blood glucose level and gluconeogenesis [85], it was found to suppress mRNA levels of phosphoenolpyruvate carboxykinase (PEPCK) and glucose-6-phosphatase (G6Pase) in the presence or absence of CDCA. In addition, in KKay mice, treatment with **6** significantly decreased fasting and non-fasting blood glucose levels. Concomitantly, **6** improved glucose tolerance and insulin sensitivity and lowered HbA1c levels [83].

In continuation of their work, Fang’s group designed and synthesized more OA hybrids with 12β-*O*-β-aspartyl or 12β-*O*-γ-glutamyl moiety trying to enhance the affinity of compound **6** towards FXR-LBD as shown for compounds **7**–**10** [83,84]. The design considered the presence of unoccupied hydrophobic space around the compound **6** terminal chain, thus aimed at using more branched chains. The authors adopted similar synthetic procedures for OA 12β-OH esterification that were used for the synthesis of compound **6,** as mentioned above. Among the new series, compound **10** with *S*-γ-glutamyl moiety possesses the highest antagonism to FXR with IC_50_ 0.44 µM in HEK293T cells. Using the *R-*γ-glutamyl chain lowered the activity (**9**, IC_50_ 1.95 µM). OA-bearing *S*-aspartyl chain, compound **7,** is slightly less active than **10** with IC_50_ 0.95 µM. Protection of the terminal amino- group by *tert*-butyloxycarbonyl (Boc) and the terminal carboxyl with *tert*-butyl (*t*-Bu) group is crucial for activity; for example, compound **8** with both free terminal groups dramatically lost activity. In general, OA derivatives bearing (*S*) configuration side chains outperform those bearing (*R*) configuration ones, and glutamyl derivatives outperform aspartyl derivatives. In a similar fashion to compound **6**, **10** showed favorable selectivity against FXR with much less effect on LXRα and PPARα and no effect on other tested metabolic NRs. Consequently, **10** significantly inhibited the expression of PEPCK and G6Pase at 1 and 10 µM concentrations. In hepatic stellate cell line LX-2, compound **10** lowered mRNA expressions of liver fibrosis marker genes, including collagen type I α-1, actin-α-2, transforming growth factor β-1, connective tissue growth factor, and integrin α-V, whereas guggulsterone lowered expression of only collagen type I α-1, actin-α-2.

In a bile duct ligation (BDL) rat model, the oral administration of compound **10** for two weeks effectively decreased the levels of AST, ALT, TBA, and TBiL, which means less liver fibrosis. Liver histopathology using hematoxylin and eosin (H&E) staining showed that compound **10** intake alleviated bile duct hyperplasia and parenchymal necrosis that accompany BDL. The collagen-specific Sirius red staining showed less collagen accumulation in the compound **10** treated group. These in vitro data of reducing hepatic fibrosis markers were further validated in vivo. Interestingly, in a NASH mice model, the titled compound reduced intrahepatic steatosis and hepatic lobular inflammation, indicating less liver fat accumulation. This is accompanied by a reduction in mRNA expression of fibrosis marker genes. Collectively, compound **10** is an OA derivative and a promising FXR modulator with NASH and diabetes therapeutic potential [84].

Collectively, OA reprograms the liver to protect against hepatotoxic chemicals, but its intake should be with care since its high doses are reported to be hepatotoxic and can develop cholestatic liver injury [41,86,87]. It is worth noting that the knockdown of FXR ameliorated OA-induced cholestatic liver injury [86]. Such paradoxical hepatoprotection and hepatotoxicity are common for natural herbs. In conclusion, OA dose is the one to differentiate between its role as a remedy and a poison [88].

### 3.2. Modulation of PPARs

PPARs comprise three different subtypes, α, β, and γ, that orchestrate lipid homeostasis and insulin sensitivity, making them attractive targets for controlling metabolic syndrome and diabetes [89,90,91,92]. PPARα reduces the formation of triglycerides; however, PPARβ controls serum lipid profile and insulin sensitivity [93]. PPARγ has a significant role in controlling insulin sensitivity and adipogenesis [94]. Fibrates and thiazolidinedione are two classes of PPARs modulators approved for hyperlipidemia and diabetes therapy, respectively [19]. Fibrates such as pemafibrate modulate PPARα, whereas thiazolidinediones such as rosiglitazone upregulate PPARγ [95].

In 2005, Huang et al. revealed that OA is a concentration-dependent PPARα activator through luciferase reporter assay in human embryonic kidney 293 cells, unlike ursolic acid and gallic acid. The OA-induced activation of PPARα was demolished upon adding a selective PPARα antagonist MK-886 [96]. The anti-hyperlipidemia effect of Pomegranate flower extract was ascribed mainly to the presence of OA [96]. Additionally, OA is reported to be a weak activator of PPARγ, which has an in-part role in the antidiabetic activity of Salvia *officinalis* extract [97,98]. Such results contradict the reported selective modulation of FXR by OA [74].

The generation of a pharmacophore model of PPARγ partial agonists using the Chinese natural product database led to the identification of OA as a PPARγ modulator [99]. Chios mastic gum (CMG) is therapeutically beneficial in managing diabetes [100], hyperlipidemia, insulin resistance [101], and diet-induced NASH [102]. Combining regular physical exercise with CMG intake for six months highly enhanced those health benefits in young Japanese men [101]. Those health benefits were attributed to the presence of a high amount of OA in CMG and its modulation of PPARγ [100,103].

**Figure 5:**
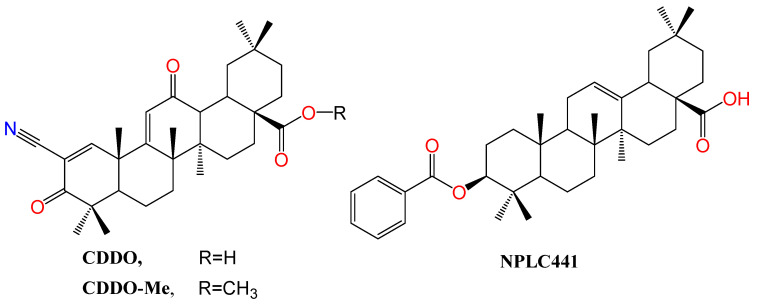
Structure of OA derivatives CDDO and CDDO-Me, PPAR modulators, and NPLC441, an RXR modulator. Atoms were colored by element.

The synthetic derivative of oleanolic acid, 2-Cyano-3,12-dioxooleana-1,9-dien-28-oic acid (CDDO) (Figure 5), is endowed with not only anticancer and anti-inflammatory activity but also partial agonistic PPARγ agonism. Intriguingly, its methyl ester, called CDDO-Me, is reported as a PPARγ antagonist [104]. PPARγ binding competition in the presence of rosiglitazone using scintillation proximity assay (SPA) showed that the ki values for binding to PPARγ are 12 nM and 130 nM for CDDO and CDDO-Me, respectively [104]. As evidence of selectivity, neither of them could interact with PPARα.

### 3.3. Modulation of LXR

LXR (α,β) are naturally activated by oxysterols and implicated in glucose homeostasis, lipid homeostasis, cancer, atherosclerosis, and NASH [16,105]. LXR plays a crucial role in regulating hepatic de novo lipogenesis (DNL) and cholesterol homeostasis [106]. LXRα activation promotes the expression of hepatic lipogenic genes through the activation of SREBP-1c2. Hepatic expression of LXR is elevated in non-alcoholic fatty liver disease (NAFLD) patients; hence, antagonizing LXR may be of therapeutic benefit [107,108]. On the other side, synthetic LXR agonists such as compound T090 are anti-atherosclerotic agents, albeit with severe undesirable effects, including elevated de novo lipogenesis and consequent development of hepatic steatosis. Both LXR subtypes share over 70% amino acid identity and architecture with PPARs.

OA was identified as an LXR antagonist, which is similar to the effect of its congener ursolic acid [55,109]. In differentiated HepaRG, hepatocyte-like cells, OA reversed the T090-indued lipid accumulation and elevation of mRNA expression and protein level of the SREBP-1c promoter in a dose-dependent manner. In other words, OA downregulated the mRNA and protein levels of target genes involved in the LXRα-SREBP-1c signaling pathway. OA activity is observed in HepaRG but not in colon cells LS174T, confirming the cellular-context paradox. OA fitted snugly in LXRα-LBD, adopting a similar orientation of T090 as calculated by molecular modeling [109].

On the contrary, in rats, another cellular model, OA oral administration promoted mRNA expression and protein levels of liver detoxification enzymes, including hydroxylation, glucuronidation, sulfation, and glutathione conjugation enzymes. This helped protect the liver from bile acids-induced toxicity in BDL rats. OA hepatoprotective effect is basically attributed to increasing the mRNA expression of LXRα and other transcription factors. At the protein level, not only LXRα was elevated but also PXR, RAR, and VDR [43].

### 3.4. Modulation of RXR

RXR, with three subtypes (α,β,γ), are modulated by rexinoids and represent a new avenue in immunomodulation for battling inflammatory diseases, cancer, and other diseases [110,111]. As mentioned above, some NRs must dimerize with RXR to start their transcriptional function. NPLC441 (Figure 5), an OA derivative, promoted LXRα:RXRα heterodimer transactivation in HEK293 cells using a luciferase reporter assay in a dose-dependent manner, unlike the parent, OA, which lacked this activity. NPLC441 was unable to bind LXRα-LBD while it competed with 9-cis-retinoic acid for binding with RXRα-LBD. Therefore, NPLC441 elicits its action on LXRα:RXRα heterodimer by binding to RXRα-LBD solely with a KD value of 0.72 µM, which highly outperforms OA, KD value of 321 µM. Surprisingly, NPLC441 could not activate other NR heterodimers, including PPARγ:RXRα and FXR:RXRα. Concomitantly, NPLC441 LXR-dependent expression of ATP-binding cassette transporter A1 (ABCA1) and ABCG1. Treatment of 3T3-L1 adipocytes with NPLC441 elevated insulin-regulated glucose transporter 4 (GLUT4) gene transcription in a dose-dependent manner and increased cellular glucose uptake. GLUT4 is a crucial regulator of insulin-regulated glucose uptake into fat and muscle cells [112]. Finally, the authors indicated that NPLC441 suppresses 11β-Hydroxysteroid dehydrogenase type 1 (11β-HSD1) expression in HepG2 cells, which is mediated by LXRα:RXRα activation, not by GR modulation [16]. Away from NRs modulation, both OA and NPLC441 are PTP1B inhibitors, making them beneficial for diabetes type-2 management [16,47].

### 3.5. Modulation of PXR and CAR

PXR controls metabolism, detoxification, and clearance of xenobiotics from the body as they, alongside CAR, have a pronounced role in regulating cytochrome P450 (CYP 450) expression, including the two major types CYP3A4 and CYP2B6 [113,114]. Additionally, PXR has a prominent role in lipid metabolism, liver health, and glucose homeostasis [115,116]. PXR is activated by ubiquitous endogenous and exogenous ligands, including steroids, bile acids, antimycotics such as clotrimazole, and antibiotics such as rifampicin [117]. Despite their considerable role in metabolism, PXR and CAR overactivation is sometimes accompanied by certain types of drug-induced cytotoxicity, such as that of paracetamol and isoniazid.

OA was identified as a PXR/CAR modulator that competes with the strong agonist rifampicin. In this regard, the possible increase in CYP3A4-mediated drug metabolism by rifampicin could be reversed by OA, which reduced the inducible forms of CYP3A4 and CYP2B6 mRNA and protein in the presence of rifampicin. OA significantly attenuated rifampicin-isoniazide-induced cytotoxicity and enhanced glutathione concentration HepaRG Cells at ten µM in comparison with OA-untreated cells [118]. Hence, OA may efficiently act to minimize undesirable interactions between transcriptional inducers of CYP450 and co-administered drugs [118].

Interestingly, Lin et al. assessed the OA effect on PXR transcriptional activation of genes implicated in lipogenesis, including S14 and SCD. A reporter assay showed that activation of promoters S14 and SCD by rifampicin could be efficiently reversed by OA. mRNA and protein expression of S14, FAS, SCD, ACC, ACLY, and FAE genes was reduced by OA even in the presence of rifampicin. HepaRG cells staining Oil Red O and observation by phase-contrast microscope revealed rifampicin-induced lipid accumulation, which was remarkably reduced by treatment with OA [109].

### 3.6. Modulation of ROR

ROR controls Th17 lymphocyte differentiation, which, in turn, secrets interleukins (IL) that fight pathogens. However, the overactivation of Th17 cells is observed in different autoimmune disorders such as multiple sclerosis, psoriasis, and rheumatoid arthritis, which confers a potential role in controlling these diseases to ROR modulation [119]. Of interest, the RORγt subtype is only expressed in immune cells, particularly Th17 [54,119]. Pastwinska et al. used cheminformatics to identify OA, alongside ursolic acid and corosolic acid, as potential RORγt inverse agonists by binding to it LBD. In silico results were validated by different approaches. In HEK293 cells, OA reduced RORγ reporter activity in a dose-dependent manner. Consistently, RORγt-dependent expression of IL17A and IL17F was diminished in Th17 cells. Chromatin immunoprecipitation showed that oleanolic acid perturbs the binding of RORγt to the promoters of the IL17A and IL17F genes [119]. This may be a clue for using OA for autoimmune disorders therapy.

## 4. Conclusions and Future Directions

Finding selective modulators for NRs with efficient health benefits and minimal deleterious effects is like finding a needle in a haystack and is a real challenge in the hard-fought battle against metabolic syndromes that are directly related to heart and cardiovascular disease. Befitting their pivotal role in physiological homeostasis, and also their druggability, we aimed to review NRs with respect to modulation by one of the most prominent phytochemicals, OA. Indeed, OA demonstrated pronounced pharmacodynamic activities against metabolic NRs, holding a clinical promise for the treatment of a batch of metabolic disorders, including NASH, diabetes, and atherosclerosis. It was normal to witness varying effects of OA on NRs, which are cell- and gene-type-specific, confirming the importance of considering the cellular context of each NR target. So far, a few OA derivatives have been semi-synthesized and benchmarked against NRs; hence, screening of more rationally designed derivatives is still required and may open the way for the discovery of selective NRs modulators.

## Data Availability

Not applicable.
